# Brain Gray Matter Volume and Functional Connectivity Are Associated With Smoking Cessation Outcomes

**DOI:** 10.3389/fnhum.2019.00361

**Published:** 2019-10-15

**Authors:** Wei Qian, Peiyu Huang, Zhujing Shen, Chao Wang, Yihong Yang, Minming Zhang

**Affiliations:** ^1^Department of Radiology, The Second Affiliated Hospital, Zhejiang University School of Medicine, Hangzhou, China; ^2^Neuroimaging Research Branch, National Institute on Drug Abuse, National Institutes of Health, Baltimore, MD, United States

**Keywords:** gray matter, magnetic resonance imaging, nicotine addiction, smoking cessation, functional connectivity

## Abstract

Smoking cessation is critical for reducing the risk of respiratory, cardiovascular diseases and cancers. However, most cessation attempts resulted in failure. In the present study, we aim to explore whether alterations of brain gray matter (GM) volume and functional connectivity (FC) are related to cessation outcomes, in hope of providing evidence for improving smoking cessation outcomes. Seventy-three smokers and 41 non-smokers were enrolled in the present study. All smokers participated in a 12-week smoking cessation treatment during which Varenicline was used to aid cessation. At the end of treatment, the smokers were divided into quitters and relapsers based on their abstinence performance. Structural magnetic resonance imaging (MRI) and voxel-based morphometry were applied to quantify the differences of regional brain volumes among the three groups at baseline. In addition, resting-state FC was used to investigate the related functional changes. In comparison with non-smokers, the smokers showed smaller GM volume in the left dorsal medial thalamus. Among the 73 smokers, 29 subjects successfully quitted smoking. The quitters showed greater GM volume than the relapsers in the right postcentral gyrus, right putamen\caudate nucleus and left orbitofrontal cortex (OFC). The GM volume in the left OFC was found to be negatively correlated with the pack years and daily smoking amount in the quitters. Furthermore, we found significantly reduced FC between left thalamus and left cerebellum in the relapsers. These findings extended our knowledge of the neural mechanism of smoking cessation, and suggested that brain structural and functional changes were related to smoking cessation outcomes.

## Introduction

Chronic cigarette smoking is a risk factor for respiratory, cardiovascular diseases and cancers (Ezzati and Lopez, 2003). Smoking cessation is the most direct and effective way to reduce its harm. However, a majority of smoking cessation attempts failed (Yang et al., 2005), due to nicotine addiction. Understanding the addiction mechanism, as well as identifying characteristic brain features related to successful quitting, are critical for improving cessation outcomes.

Brain structure is the foundation of brain functions and behaviors, including addiction. Magnetic resonance imaging (MRI) has been widely used to explore the brain morphometry differences between smokers and non-smokers and has become an important method to identify neural changes related to nicotine addiction. Gray matter (GM) volume and/or density changes have been found in smokers in multiple regions, including the prefrontal cortex (PFC; Brody et al., 2004; Gallinat et al., 2006; Almeida et al., 2008; Zhang et al., 2011a,b; Liao et al., 2012; Morales et al., 2012), anterior cingulate (Gallinat et al., 2006; Liao et al., 2012), thalamus (Gallinat et al., 2006; Almeida et al., 2008; Liao et al., 2012), temporal lobe (Gallinat et al., 2006), cerebellum (Gallinat et al., 2006; Yu et al., 2011; Kühn et al., 2012), etc. Alterations in these regions may contribute to the impairment of various cognitive functions (Ullsperger and von Cramon, 2001; Grecucci et al., 2013; Wang et al., 2016), and lead to addictive behaviors.

Furthermore, studies on a variety of diseases showed that the integrity of brain structure is tightly coupled with brain functional alterations. Zhang et al. (2011b) combined the structural (GM) and functional MRI (fMRI) data in a nicotine addiction study. They found that smokers had lower GM density in the dorsolateral PFC (dlPFC), and decreased resting-state functional connectivity (FC) between the dlPFC and rostral anterior cingulate gyrus. Another multi-modal study showed that smokers had lower GM density in the anterior insula and decreased connectivity between the anterior insula and PFC (Stoeckel et al., 2016). Previously, we also demonstrated that smaller GM volume in the Crus I of cerebellum of smokers was related to decreased FC between the Crus I and several brain regions related to cognitive and motor functions. The coupling of structural and functional alterations could reinforce the circuitry changes and is a possible reason for relapse.

Although brain structure and FC have been consistently linked to nicotine addiction, it remains unclear whether they are associated with smoking cessation, which is of great clinical importance. Our previous fMRI studies measured the regional spontaneous brain activity using Regional homogeneity (ReHo) and found decreased ReHo in the bilateral posterior cingulate cortex and increased ReHo in the left superior temporal gyrus were associated with worse smoking cessation outcomes (Wang et al., 2017). In addition, two diffusion tensor imaging studies found that white matter integrity was also different in smokers successfully quitted or relapsed, encompassing striato-cortical tracts (Yuan et al., 2018), and fibers adjacent to the cerebellum and postcentral gyrus (Huang et al., 2017). As mentioned above, brain GM plays an important role in smoking addiction, but there are few studies investigating its relationship with cessation outcomes. Only one study demonstrated in a 4-week longitudinal study that the successful quitters had greater GM volume in the right occipital lobe and left putamen, but smaller GM volume in the right cuneus and bilateral hippocampus (Froeliger et al., 2010). While this study implicated the possible relationship between GM volume and smoking cessation, these findings were limited by its small sample size (total *n* = 18). Besides, it is unclear whether GM alterations would cause further functional network disturbances and strengthen addiction behaviors. These questions can only be answered by combining information from multi-modality images.

In the present study, we are interested in how brain morphometry characteristics and FC differences contribute to smoking cessation attempts. High-resolution structural MRI and voxel-based morphology (VBM) analysis were adopted to study the brain GM volume differences between smokers and non-smokers, and to detect whether baseline GM differences were related to Varenicline treatment outcome in smokers. Moreover, FC was employed to further explore communications of brain regions related to the structural alterations. We expected to see that smokers with different cessation outcomes might have different GM volume and FC strength at baseline, especially in brain regions within the reward circuit and cognitive control circuit.

## Materials and Methods

### Participants

A total of 126 subjects participated in this study, enrolled through public posters and online forms. Eight cigarette smokers (five of them were considered as quitters and three of them were considered as relapsers after a 12-week smoking cessation treatment) and four non-smokers were excluded owing to inordinate head motions during MR scanning, resulting in 114 subjects (73 cigarette smokers and 41 healthy non-smokers). Smokers were defined as those who had smoked at least 10 cigarettes per day for more than 2 years and met the criteria for nicotine dependence in Diagnostic and Statistical Manual of Mental Disorders, 4th Edition (DSM-IV), had an afternoon expired CO level ≥10 ppm. Expiratory carbon monoxide (CO) levels of all subjects were measured using the Smokerlyzer System (Bedfont Scientific, Limited, Rochester, UK; Yuan et al., 2018). The subjects were excluded for any lifetime history of neurological or psychiatric diseases, such as brain tumor, brain traumas, depression, and other substance use disorders (except nicotine), etc. Subjects were also excluded if they had severe systemic diseases that could affect the brain, or had MRI contraindications (i.e., metal implants, claustrophobia). Non-smokers were defined as those who had smoked less than 10 cigarettes in the whole life and none in the past 10 years, had an afternoon expired CO level ≤6 ppm. The exclusion criteria were the same as for the smokers. Due to the scarcity of female smokers in China, we only recruited male smokers and healthy non-smoker controls in the present study. All participants were right-handed, 18–55 years old and Han ethnic. All subjects had signed informed consents at enrollment. The study was approved by the Institutional Review Boards of the Second Affiliated Hospital of Zhejiang University School of Medicine. Demographic (age, gender, education years) and baseline smoking data [smoking years, age of smoking initiation, daily smoking amount, Smoking index = (smoking years)* (cigarettes per day)] were collected from all participants by a questionnaire before MR scanning. All smokers were assessed using Fagerström Test for Nicotine Dependence (FTND, Heatherton et al., 1991) to measure the nicotine dependence severity.

### Procedures

An MRI scanning session at baseline was performed in all subjects. All the smokers were allowed to smoke cigarettes before MR scanning. In other words, they were scanned at satiated state. Then, all smokers started a 12-week drug-aided smoking cessation treatment. Varenicline (tartrate, trade name Champix, Pfizer manufactured, imported drug registration No. H20080417 and H20080416) was used following the instructions (FDA treatment manual^1^) as adjuvant therapy. Participants started with a recommended varenicline dosage of 0.5 mg QD for the first 3 days, increasing to 0.5 mg bid for days 4–7, and following to the maintenance dosage of 1 mg bid. Since the beginning of the treatment, weekly telephone follow-ups were conducted to evaluate the smoking status (including smoking cessation processing, adverse effects, etc.) and to provide medical coaching and counseling during the study participation.

### Smoking Cessation Outcome Evaluation

The endpoint was the 4-week continuous abstinence for the last 4 weeks of drug therapy (weeks 9–12). The condition of abstention was defined by self-reported abstinence and biological verification of the CO ≤6 ppm.

### Image Acquisition

Studies were performed on a 3.0 Tesla GE Signa MR system using a standard phased-array head coil. Resting-state blood oxygen level dependent (BOLD) images were collected with the echo-planar imaging (EPI) sequence: 30 slices (thickness/gap = 4/1 mm), repetition time (TR) = 2,000 ms, echo time (TE) = 30 ms, field of view (FOV) = 240 × 240 mm^2^, matrix = 64 × 64, flip angle = 80°. A total of 185 whole brain images were acquired, during which the participants were instructed to stay calm and awake with their eyes closed. Anatomical images were collected with the high-resolution fast spoiled gradient recalled (FSPGR) sequence: 136 slices, 1 mm thickness, TR = 5 ms, TE = 1.1 ms, TI = 400 ms, FOV = 24 × 24 cm^2^, matrix size = 240 × 216 mm, flip angle = 15°. Several other scans were acquired, which were not directly related to data used in this study, and the total scan time for each subject was about 40 min.

### Voxel-Based Morphometry

We used Statistical Parametric Mapping (SPM8^2^) based on the Matlab platform^3^ to analyze the structural images. T1 weighted images were firstly separated into different tissue types, including gray, white matter, cerebrospinal fluid (CSF), etc. Then, the segmented GM images were used to create study-specific group templates to achieve accurate inter-subject registration using Diffeomorphic Anatomical Registration using Exponentiated Lie algebra (DARTEL; Ashburner, 2007). This algorithm is to create a group-wise brain template by iteratively aligning each individual’s tissue maps to the group template. Next, we normalized each participant’s brain into MNI space with the normalized images modulated to make sure that relative GM volumes were maintained following spatial normalization. Finally, modulated GM images were smoothed with a 4 mm FWHM Gaussian kernel for statistical analyses.

### Pre-processing of Resting-State fMRI Images

Pre-processing was implemented with Data Processing Assistant for Resting-State fMRI (DPARSF, Yan and Zang, 2010), by YAN Chao-Gan) and Resting-State fMRI Data Analysis Toolkit [REST (Song et al., 2011), V1.8^4^]. The first 10 images were excluded to allow the magnetization to reach a steady state. We used the middle slice for reference to correct the slice timing of surplus images and realign to eliminate head motion. Several nuisance covariates, including the Friston-24 head motion parameters (Friston et al., 1996), CSF and white matter signals (calculated with the compcor method (Behzadi et al., 2007), within the mask from the segmented co-registered T1 images), were regressed out to increase signal-noise ratio and reduce the motion artifact. The resulted images were normalized into the standard space through T1 images following DARTEL procedures and resampled to 3 × 3 × 3 mm voxel size. Then we smoothed the images using a 6-mm Gaussian kernel, removed the linear trend, and performed bandpass filtering (0.01–0.1 Hz).

During pre-processing, subjects who had a head motion with >2 mm of translation or 2° of rotation were excluded to further minimize the influence of head motion on FC estimation. Framewise displacement (FD, Power et al., 2012) of head motion was evaluated with the sum of the absolute values of the six motion parameter derivatives. Using FD, we censored each subjects’ resting-state BOLD time series that were associated with occasional head motion and excluded the time point where FD >0.5 mm. After this scrubbing procedure, subjects whose total length of data were <130 were also excluded. Twelve subjects were excluded because of excessive head motion in the present study.

### Statistical Analysis and Functional Connectivity Analysis

One-way analysis of variance (ANOVA) was applied to analyze the difference of age and education years among the three teams. Two-sample *t*-tests was conducted to evaluate group differences between the quitters and relapsers.

For image analysis, ANOVA and two-sample *t*-tests were conducted in SPM to examine the differences of GM volume among different groups, controlled for age and education years. The analysis was constrained in a GM mask generated by applying a threshold to the group averaged GM template. To demonstrate brain GM alterations in smokers, we pooled the relapsers and quitters into one group as smokers and then compared their brain GM volume with the non-smokers. The statistical threshold for VBM analysis was set at voxel-level *p* < 0.001. Multiple comparison correction was performed at the cluster level using Gaussian Random Field (GRF) theory implemented by SPM, resulting in a corrected *p* < 0.05.

After the whole brain voxel-based analysis, we used the significant clusters to construct regions of interest (ROIs) and extracted mean values from the modulated brain images. Then we calculated partial correlations between regional brain volumes (from five ROIs) and smoking-related behavioral scores (five indices as shown in Table 1), with age and education used as covariates. A total of 25 correlation analysis was performed, and we used false discovery rate methods to reduce type I errors.

**Table 1:** Demographic characteristics of the participants.

	Mean (SD)			Test value	*P*-value
	Non-smokers	Relapsers	Quitters		
Age (years)	38.5 ± 7.4	39 ± 6.5	38 ± 7.3	0.160	0.852
Education (years)	15.4 ± 4.7	13.7 ± 2.6	14.1 ± 2.8	2.390	0.096
Age of smoking		20.1 ± 4.5	21.9 ± 6.2	−1.412	0.162
Smoking years		18.9 ± 6.4	16.1 ± 6.6	1.781	0.079
Cigarettes per day		23.6 ± 10.4	22.8 ± 9.2	0.335	0.739
Smoking index		445.5 ± 255.8	377.5 ± 233.9	1.149	0.254
FTND		5.4 ± 2.4	5.2 ± 1.9	0.320	0.750

FTND, Fagerstrom Test of Nicotine Dependence; Smoking index: (smoking years)* (cigarettes per day).

We also used these ROIs as seed regions to perform FC analysis. Pearson correlations between the mean BOLD signal of each ROI and signals from other voxels in the brain were calculated to create FC maps. Then, differences of FC maps among the three groups were assessed using one-way ANOVA, controlled for age and education years. The voxel-level threshold was *p* < 0.001 and the cluster-level threshold corrected for multiple comparisons (using GRF method) was *p* < 0.0124, as four correlation analysis had been performed. *Post hoc* analysis was performed to demonstrate the differences.

## Results

### Demographic Information

The characteristics of all the subjects are provided in Table 1. The three groups did not have significant differences in age or education level. Among 73 smokers, 29 subjects who met the abstinence criteria were considered as quitters, and the other 44 subjects were considered as relapsers. There were no significant differences between quitter and relapser groups on any demographic characteristics or smoking features.

### GM Volume Differences Between Non-smokers, Quitters and Relapsers

**Figure 1:**
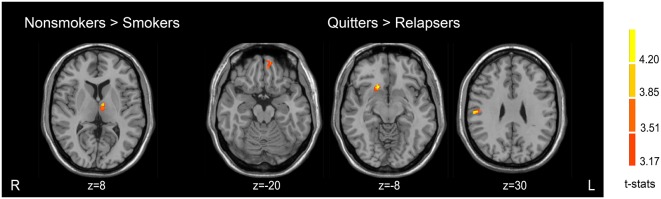
Brain regions showing different gray matter (GM) volumes among the three groups.

There were GM volume differences between the three groups in the left dorsal medial thalamus, right postcentral gyrus, right putamen and caudate nucleus (dorsal striatum), and left orbitofrontal cortex (OFC). Compared with non-smokers, relapsers showed decreased GM volume in the left dorsal medial thalamus. No significant result was found in the non-smokers-quitter comparison. Quitters demonstrated greater GM volume in the right postcentral gyrus, right putamen and caudate nucleus (dorsal striatum), and left OFC than relapsers. Detailed results were presented in Table 2 and Figure 1. These clusters showing significant differences were then used as ROIs for further analyses.

**Table 2:** Brain regions showing significant gray matter (GM) volume differences among the three groups.

Region	Cluster size (No. Voxels)	Coordinates MNI (mm)			F_max_/T_max_	Effect size^#^
		*X*	*Y*	*Z*		
**ANOVA analysis**
L orbitofrontal cortex	50	−4	58	−16	11.83	0.51
R putamen nucleus\caudate nucleus	39	18	20	−8	10.20	0.50
L thalamus (dorsal medial)	36	−8	−8	8	9.996	0.33
R postcentral gyrus	41	58	−18	28	10.36	0.49
**Non-smokers vs. Quitters**	No result					
**Non-smokers vs. Relapsers**						
L thalamus (dorsal medial)	49	−8	−8	8	4.44	0.35
**Quitters vs. Relapsers**
R postcentral gyrus	35	58	−29	30	4.54	1.18
R putamen nucleus\caudate nucleus	48	18	20	−8	4.50	1.18
L orbitofrontal cortex	40	−10	54	−20	4.18	1.21
**Non-smokers vs. Pooled Smokers**
L thalamus (dorsal medial)	35	−8	−8	8	4.01	0.37

*R, right hemisphere; L, left hemisphere; MNI, Montreal Neurological Institute. #, Cohen’s f for ANOVA analysis; Cohen’s d for two-sample *t*-test*.

### GM Volume Differences Between Smokers and Non-smokers

Smokers revealed smaller GM volume in the left dorsal medial thalamus compared with non-smokers. There was no region with greater GM volume in smokers. The detailed results of the VBM analysis are showed in Table 2 and Figure 1.

### ROI Analysis

**Figure 2:**
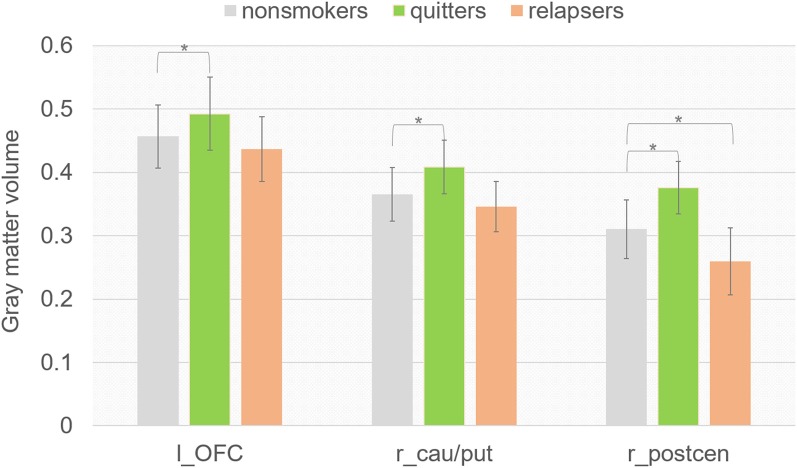
Mean regional values extracted from modulated GM images using ROIs where quitters differed from relapsers.

To better understand the GM volume difference between quitters and relapsers, we compared the GM volume in the three clusters where quitters differed from relapsers between non-smoker group and each smoker groups. Interestingly, we found (Figure 2) that quitters had significantly higher GM volume in all three clusters than non-smokers (left OFC, *p* = 0.003; right caudate/putamen, *p* = 0.002; right postcentral gyrus, *p* = 0.015), and relapsers had significantly lower GM volume in the right postcentral gyrus than non-smokers (*p* = 0.032). The GM volume in left OFC was found to be negatively correlated with the cigarettes per day (*r* = −0.525, *p* = 0.012, uncorrected) and smoking index [(smoking years)* (cigarettes per day), *r* = −0.503, *p* = 0.017, uncorrected] in quitters, but not in relapsers. However, the correlation findings did not survive multiple comparison correction. GM volume in other ROIs did not show significant correlation with any of the smoking-related behavioral index.

### Functional Connectivity Analysis

**Figure 3:**
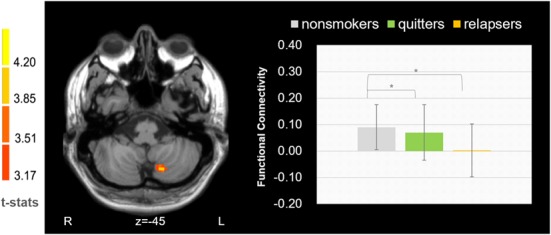
Functional connectivity between the left dorsomedial thalamus and cerebellum was found decreased in relapsers.

We found that the FC between the left dorsal medial thalamus and left cerebellum (Figure 3, cerebellum lobule VII, cluster size = 34, MNI coordinate: −15 −69 −45, *p* = 0.002) showed significant difference among the three groups. *Post hoc* analysis revealed that the difference was driven by the decreased connectivity in relapsers compared with non-smokers (*p* = 2.11*10^−4^) and quitters (*p* = 0.022). No correlation was found between thalamus-cerebellar-FC and behavioral scores.

## Discussion

In the present study, we investigated the GM volume and FC differences between the smokers and non-smokers, and their relationship to smoking cessation. We found that smokers had smaller GM volume in the left dorsal medial thalamus than non-smokers. Quitters had greater GM volume in the right postcentral gyrus, right putamen/caudate nucleus (dorsal striatum), and left OFC than relapsers and non-smokers. In addition, FC between the left dorsal medial thalamus and cerebellum is significantly decreased in the relapser group.

The observed volumetric decrease in the thalamus of cigarette smokers is in congruence with several prior studies (Gallinat et al., 2006; Almeida et al., 2008; Liao et al., 2012; Peng et al., 2017). Recently, Sutherland et al. (2016) performed a coordinate-based meta-analysis of MRI studies related to chronic smoking, which suggest that thalamus is one of the structures showing consistent structural decreased in smokers. The thalamus is rich in nicotinic acetylcholine receptors (nAChRs; Cecilia et al., 2006), which have a high affinity to nicotine. Therefore, chronic smoking may induce long term synaptic changes in thalamus. Previous studies revealed that thalamus participates in several cognitive brain functions, including inhibitory control, arousal regulation, sustained attention and others. Smaller thalamus volume may be related to the disruption of these cognitive function in smokers. In fact, Froeliger et al. (2017) showed that weaker corticothalamic-mediated inhibitory control seemed to be related to sooner relapse in smokers. Therefore, it is likely that the smaller thalamus volume found in the current study may indicate impaired cognitive functions in smokers.

In addition to the volumetric changes, we found decreased FC between the left dorsomedial thalamus and left cerebellum (lobule VII) in relapsers as well. Although, cerebellum is usually considered as a structure for motor control, recent brain imaging studies demonstrate that it also involves in cognitive functions (Stoodley, 2012). Increasing studies show that lobule VII plays an essential role in working memory, executive functions and emotional processing (Stoodley and Schmahmann, 2009). The lobule VII involves in nicotine addiction possibly by taking part in the constitution of habitual behaviors. Repeated drug intake may re-organize the relevant cortical-cerebellum circuitry, reinforcing drug-related memories, anticipations and motivation behaviors (Miquel et al., 2009). Furthermore, altered lobule VII GM volume and function have been coincidentally reported in smokers (Kühn et al., 2012; Franklin et al., 2014). Interestingly, the dorsomedial thalamus has connections with both frontal lobe and cerebellum, and is considered an important relay node for cerebral-cerebellar interactions (Yamamoto et al., 1992). It is possible that the decreased thalamus-cerebellar FC may have hindered the communication between frontal lobe and cerebellum, invalidating the top-down regulations. Therefore, the relapsers may experience difficulties in utilizing cognitive abilities to reverse habitual behaviors.

The study also found that the quitters had larger OFC volume prior to smoking cessation. The OFC is a crucial node of the brain reward system. It exerts top-down regulation to regions that are related to emotional responses and reward processing. Impairments of the OFC may result in enhanced stress reactivity and inability to contain emotional moods (Bechara et al., 2000), increased intention to seek drugs (Rolls, 2000), and poorer ability to restrain drug-cue related behaviors (Volkow and Fowler, 2000), all of which could lead to substance addiction. Cortical thinning in the OFC has been reported in young male smokers, suggesting the contribution of fronto-striatal damage to smoking addiction (Dom et al., 2005). Besides, a large number of fMRI studies have found that smoking-related cues can increase the activation of the OFC. There has been a consensus that OFC is involved in substance addiction, although its relationship with smoking cessation is still unexplored. Our findings suggest that the structural integrity of OFC is important for quitting smoking.

We also revealed that better smoking cessation outcomes were related to greater right putamen and caudate nucleus (dorsal striatum) GM volume at baseline. The dorsal striatum plays a vital role in the Nigrostriatal pathway, which innervated by dopaminergic neurons of the substantia nigra (Volkow et al., 2006). It also contains cholinergic interneurons, which release acetylcholine and has a key role in regulating striatal and basal ganglia functions (Gonzales and Smith, 2015). It supports a variety of functions including habit learning and automaticity (Everitt and Robbins, 2013). The role of dorsal striatum in substance abuse has been broadly recognized (Everitt and Robbins, 2013). Smaller dorsal striatum GM volume may be associated with a disturbance of DA functions, and it may contribute to the neurobiology of nicotine abstinence symptomatology. There are some fMRI studies indicating that responses in the dorsal striatum to drug-related cues may predict cessation outcomes (Wilson et al., 2014; Sweitzer et al., 2016), including a variety of substances [e.g., cannabis, cocaine, and methamphetamine (Martinez et al., 2011; Wang et al., 2012; Cousijn et al., 2013)].

Furthermore, we investigated the correlation between the regional brain volume and smoking-related behaviors. The correlation analyses showed that cigarette per day and smoking index were correlated with GM reduction in the left OFC in quitters. The other ROIs did not show significant correlation with any of the behavioral data. Zhong et al. (2016) found that smokers showed more GM atrophy in PFC with longer smoking years and more cigarette smoking per day. In our study, however, the negative correlation was found only in the quitters. Considering the interesting fact that the quitters’ OFC volume was even larger than non-smokers, this negative correlation may suggest that larger OFC volume is a resistant factor for cigarette smoking and relapse.

Understanding the factors related to smoking cessation is vital to help improve cessation outcomes. In general, lower nicotine dependence, fewer daily smoking amount, less craving in the early stage of abstinence, and high self-efficacy are most commonly repeatable clinical predictors for smoking cessation, while other predictors including strong desire to quit, few side effect, low anger, without history of depression, slow nicotine metabolism, easier reducing smoking overtime and no lapses during the early period of treatment (Brody et al., 2014). In addition to these psychological and behavioral factors, objective assessment of brain alterations may also provide useful information. Researches have been striving to find brain structural and functional markers, mostly utilizing fMRI methods. Amygdala response to smoking cues (Janes et al., 2010; Perkins, 2012) and cessation information (Chu et al., 2014) in smokers was predictive for quitting. Likewise, brain activation to emotional pictures had relation to smoking cessation success rate (Versace et al., 2014). Recently, several studies also showed that white matter integrity and regional spontaneous brain activity were associated with cessation outcomes (Huang et al., 2017; Wang et al., 2017; Yuan et al., 2018). Our present study combined the GM volume analysis and its related FC alterations to investigate the brain structural and functional characteristics with smoking cessation outcomes. The brain areas showing differences between relapsers and quitters were associated with reward, mood and behavioral control, corroborating the findings from behavioral studies. These brain regions may need to be specifically modulated to increase the likelihood of successful cessation in smokers showing similar brain structural and functional patterns.

Some limitations of the current research should be pointed out. First, it was a cross-sectional analysis, we were unable to infer whether these structural and functional changes were predisposing traits or effect of substance consumption. On the one hand, individual’s brain circuit formation was shaped by their genetic and neurodevelopment background (Li et al., 2017). On the other hand, long-term smoking may strengthen or damage several brain circuits, causing compulsive and habitual behaviors (Huang et al., 2017). While the two groups in our study had similar total smoking amount and nicotine-dependent level, their brain responses to long-term nicotine assumption may varies. Further longitudinal studies were still required to address this question. Additionally, due to the study scope, we only used varenicline in the smoking cessation treatment. Further research including more treatment methods is warranted to confirm and extend these findings. Besides, studies are needed to validate these findings on a large independent cohort and to utilize machine-learning methods to predict suitable cessation treatment.

## Conclusion

In summation, we found reduced GM volume in the left thalamus of smokers relative to non-smokers. Furthermore, better cessation outcomes were associated with larger GM volume in the OFC, dorsal striatum and right postcentral gyrus prior to smoking cessation treatment, and stronger FC between the left thalamus and cerebellum. These findings suggest that brain structural and functional changes may have a considerable promise as predictors of smoking cessation outcomes. As different people have differently wired brain circuits, they may also have different treatment responses to a variety of cessation methods. With these knowledge, we could use machine learning (Ding et al., 2015) methods to predict individual’s response, and select the most effective way to improve their probability of successful cessation.

## Data Availability Statement

The datasets generated for this study are available on request to the corresponding author.

## Ethics Statement

The studies involving human participants were reviewed and approved by Institutional Review Boards of the Second Affiliated Hospital of Zhejiang University School of Medicine. The patients/participants provided their written informed consent to participate in this study.

## Author Contributions

WQ and PH analyzed the MRI data and wrote the manuscript. ZS and CW collected the clinical and MRI data. YY and MZ assisted with study design and interpretation of findings. All authors have contributed to and approved the final manuscript.

## Conflict of Interest

The authors declare that the research was conducted in the absence of any commercial or financial relationships that could be construed as a potential conflict of interest.

## Footnotes

^1^https://www.accessdata.fda.gov/drugsatfda_docs/label/2011/021928s025lbl.pdf

^2^http://www.fil.ion.ucl.ac.uk/spm/

^3^https://www.mathworks.com/

^4^http://www.restfmri.net
